# Exploration of the optimal pulse oximetry-derived oxygen saturation target for critically ill AECOPD patients: a retrospective cohort study

**DOI:** 10.21203/rs.3.rs-2661975/v1

**Published:** 2023-03-21

**Authors:** Xuequn Guo, Donghao Guo, Qiu Luo

**Affiliations:** Quanzhou First Hospital Affiliated to Fujian Medical University; The Chinese University of Hong Kong; Quanzhou First Hospital Affiliated to Fujian Medical University

**Keywords:** AECOPD, Blood oxygen saturation, Pulse oximetry, Intensive care unit

## Abstract

**Background:**

Appropriate levels of blood oxygen are crucial for critically ill patients. However, the optimal oxygen saturation has not been confirmed for AECOPD patients during their ICU stays. The purpose of this study was to determine the optimal oxygen saturation range target to reduce mortality for those individuals.

**Methods:**

Data of 533 critically ill AECOPD patients with hypercapnic respiratory failure from the MIMIC-IV database were extracted. The association between median SpO2 value during ICU stay and 30days mortality was analyzed by LOWESS curve, and an optimal range of SpO2(92–96%) platform was observed. Comparisons between subgroups and linear analyses of the percentage of SpO2 in 92–96% and 30days or 180 days mortality were performed to support our view further.

**Methods:**

Although patients with 92–96% SpO2 had a higher rate of invasive ventilator than those with 88–92%, there was no significant increase in the adjusted ICU stay duration, non-invasive ventilator duration, or invasive ventilator duration while leading to lower 30days and 180days mortality in the subgroup with 92–96%. In addition, the percentage of SpO2 in 92–96% was associated with decreased hospital mortality.

**Conclusion:**

In conclusion, SpO2 within 92–96% could lead to lower mortality than 88–92% and > 96% for AECOPD patients during their ICU stay.

## Introduction

The supply of oxygen to the body tissue, which could be indicated by blood oxygenation, is an indispensable condition for human survival, especially for patients with critical illnesses.^1^ A golden standard for measuring blood oxygenation is the partial pressure of oxygen(PaO2) or arterial oxygen saturation(SaO2), which could only be measured by blood gas analysis. Compared to these, pulse oximetry-derived oxygen saturation (SpO2) brings more clinical utility, especially in some developing country, as it is quick, cheap, non-invasive, and repeatable.^2^ Also, research showed that SpO2 could reflect the level of SaO2 particularly when the SaO2 is above 75%.^3^

Acute exacerbation of chronic obstructive pulmonary disease (AECOPD) is an acute worsening of respiratory syndrome including breathlessness and phlegm production in patients with COPD.^4^ When AECOPD is evaluated as life-threatening based on clinical presentations, further treatment in the intensive care unit (ICU) admission will be the optimal choice.^5,6^

Previous studies have shown a strong correlation between oxygenation and mortality, making administration of oxygen supply imperative for these critically ill patients.^7^ However, the rational oxygen target saturations that would yield the maximum survival benefit for AECOPD patients during this period have not yet been identified. It’s certainly not the case that the higher oxygen concentration, the better prognosis.^8^ Hyperoxia or hypoxia is usually accompanied by a generation of free radicals causing cellular damage, and sometimes pulmonary toxicity.^9,10^ The global initiative for chronic obstructive lung disease(GOLD) suggested that blood oxygen saturations of AECOPD patients should be maintained within 88–92% during oxygen therapies.^5^ However, this conclusion comes from a study^11^ focused on emergency transport patients, despite many studies in different countries had shown the same conclusion, none of them have focused on the group of critically ill AECOPD patients.^2,12^

The British Thoracic Society(BTS) also suggested SpO2 in 88–92% for patients with COPD or other risk factors for hypercapnia, but clearly stated that management of ventilated patients on critical care units is outside the scope of the Guideline.^13^ In the large Intensive Care Unit Randomized Trial

Handling Oxygenation Targets in the ICU (HOT-ICU), investigators found lower oxygenation targets did not result in lower mortality for critically ill patients with acute hypoxemic respiratory failure.^14^ A high-quality study conducted by Boom et al. reported that SpO2 ranging from 94–98% provided the lowest mortality for critically ill patients, and SpO2 < 94% was considered to be associated with increased mortality.^8,15^ Although samples with hypercapnic respiratory failure were excluded from this study, the higher blood oxygen saturation range suggests that appropriate oxygen saturation range during ICU stay may beyond our expectations for critically ill AECOPD patients. There is still room for the selection of an appropriate oxygen saturation target range in above research for oxygen saturations of AECOPD patients, and many scholars believe that there may exist a better scope that could bring more clinical benefits.^2,8,12,16^ All of the above demonstrates that there is currently no clear oxygen therapy guideline about oxygen saturation levels for critically ill AECOPD patients. Thus, a large clinical database was employed to explore the optimal oxygen saturation range that provides the best prognosis for those patients.

## Materials And Method

### Data description

All data were retrieved from the Medical Information Mart for Intensive Care IV (MIMIC IV) open-source clinical database(version 1.0), which contains information for more than 50000 patients who were admitted to the intensive care unit (ICU) of the Beth Israel Deaconess Medical Center from 2008 to 2019.^17,18^ The approval for extraction and usage of this database has been obtained(certification number: 35875386, author Xuequn).

**Figure 1:**
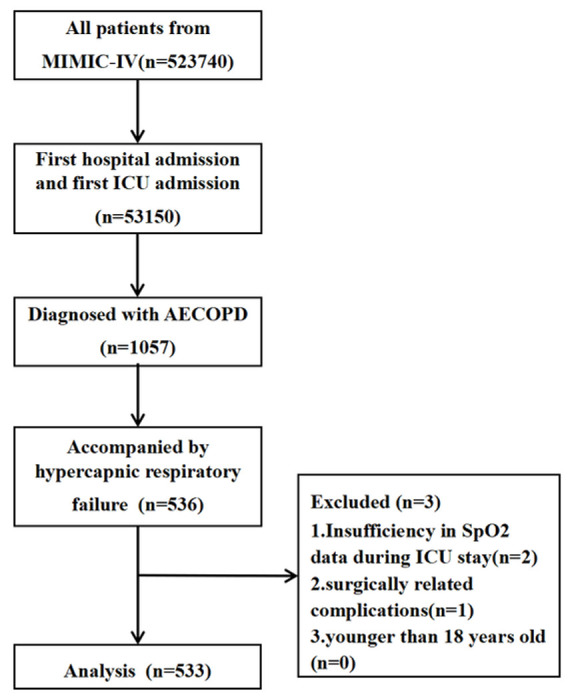
Flowchart.

The Inclusion criteria were set as follows: (1) age be equal to or greater than 18 years; (2) be diagnosed as AECOPD on admission, patients admitted with AECOPD were identified using the AECOPD ICD-10 code^19,20^; (3)to ensure that patients were admitted to ICU because of AECOPD, combined hypercapnic respiratory failure was also set as a inclusion criteria. Hypercapnic respiratory failure was defined by the max partial pressure of carbon dioxide (PaCO2) > 45 mmHg (6 kPa) within 12 hours prior to ICU admission; (4) the sufficiency of data on the pulse oximetry-derived oxygen saturation (SpO2) during ICU stay was used as a criterion to bring patients into this analysis. SpO2 is usually measured hourly in MIMIC IV. Insufficiency was defined as SpO2 recorded less than 12 times per day during ICU stay. Exclusion criteria are surgically related complications like severe trauma, burns and vital organ surgery. The primary outcome was 30days mortality with 180days mortality as a secondary outcome. Only the data of the first hospital admission and first ICU admission will be retained(Fig. 1).

Data of the demographic feature, laboratory outcomes, and disease severity scores were elicited from the database. Among them, the median of the SpO2 during ICU stay was selected as an indicator of the central trend of oxygen exposure. To allow for record deviation, only data within 2 hours deviation from ICU access time were included. Comorbidities were erected by the Elixhauser, a table created from past retrieval codes summarized in the ICD-10 codes.^19–21^ It is worth mentioning that in addition to the ICD-10 code, the use of vasopressor is also considered a prerequisite for shock. Data extraction was performed by PostgreSQL (version 10, www.postgresql.org).

### Statistical analysis

Continuous data were presented in the tables as the mean with SD or median with interquartile ranges. The Student’s t-test, Wilcoxon rank-sum test, Kruskal–Wallis test or one-way ANOVA was used as appropriate. Categorical data were presented as percentages, while the significance of the differences was determined using the Chi-squared test. The locally weighted scatterplot smoothing (LOWESS) curve, a powerful polynomial regression tool to view the nonlinear relationship between two-dimensional variables,^22^ was selected to estimate the association between median SpO2 values and short-term(30 days) mortality. By assessing trends in the curve, range of the platform with the lowest 30days mortality was harvested. The Kaplan-Meier (KM) method was used to plot unadjusted survival curves of SpO2 subgroups, and the differences among the curves were compared using the log-rank test. Univariate logistic analysis and multivariate logical analysis(stepwise regression) was used to identify the risk factors for 30days death. The relation between the percentage of SpO2 counts in optimal range and 30days or 180days mortality was displayed by LOWESS curve and further analyzed by logistic regression. Logical analysis models executed above were adjusted by age, BMI, SOFA and shock individually or together. If not specified above, a P value less than 0.05 was considered statistically significant. Stata/IC 15.1 software (StataCorp, Texas, USA) and R software (version 4.0.0, www.r-project.org) were employed for the statistical analysis.

## Results

After the implementation of inclusion and exclusion criteria, data of 533 critically ill AECOPD patients were included in the final cohort, including 88 non-survivors and 445 survivors, bringing overall mortality of 19.3% (Table 1). Compared to the survival group, The non-survival group held older age (p = 0.002), higher percentage of ICU/Hospital duration (p < 0.001), and higher SOFA scores (p < 0.001). Similarly, acidosis(p = 0.005) and shock(p < 0.001) were also higher in the non-survival group. In addition, the non-survival group received a higher oxygen supply concentration(p < 0.001) while harvesting a lower PaO2/FiO2 value(p = 0.005).

**Figure 2:**
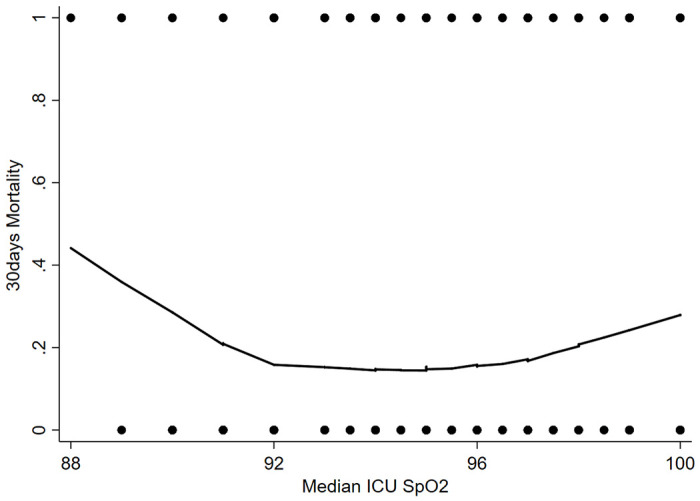
Association between median SpO2 and probability of 30days hospital mortality of critically ill AECOPD patients by LOWESS curve.

Figure 2 displayed a U-shaped association of median SpO2 values during ICU stay and 30days mortality. Since both hyperoxemia and hypoxemia are associated with a higher risk of death, the relationship between SpO2 and 30days mortality had both an upper and a lower limit, among the limits, a SpO2 flattest part with the lowest 30days mortality was observed. In the U-shape of the LOWESS curve, we can see the ascending boundary was 96%, while 92% for the descending boundary.

**Figure 3:**
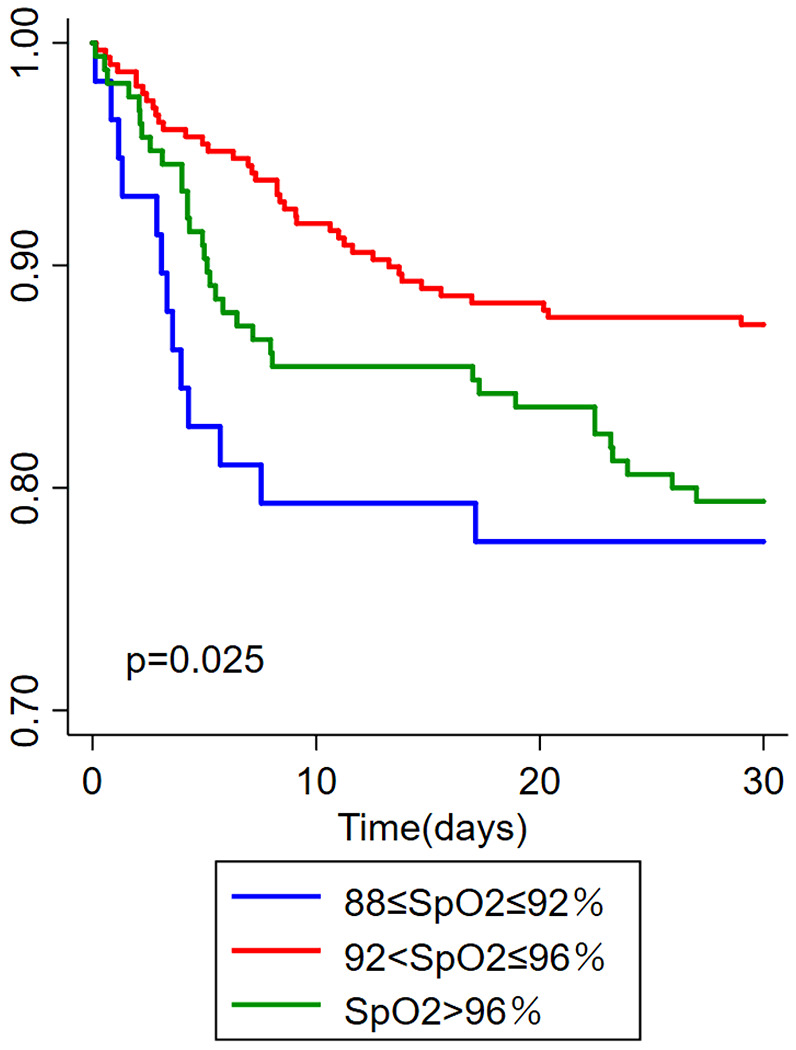
Kaplan-Meier survival curve of 30days hospital mortality for different SpOk2 subgroups(88≤SpO2≤92%, 92<SpO2≤96%, and SpO2>96%) of critically ill AECOPD patients.

According to the value of SpO2, three subgroups were set for analysis(88 ≤ SpO2 ≤ 92%, 92 < SpO2 ≤ 96%, and SpO2 > 96%). The differences in demographic feature, like age, BMI, gender, etc.., and clinical outcomes, like 30days mortality, 180days mortality, ICU duration, etc.., were compared among different SpO2 subgroups as shown in Table 2. Subgroup of 92 < SpO2 ≤ 96% had the lowest 30days and 180days mortality with an older age, a higher SOFA score, a higher shock rate, and a higher ratio of invasive ventilator(Table 2). Although the length of ICU duration was longer than 88–92%, there was no significant difference in the length of ICU duration among the three subgroups after adjusting for death. No statistically significant differences were founded in clinical outcomes among the three subgroups, including percentage of ICU/hospital duration, duration of non-invasive ventilation, and invasive ventilation. As shown in Fig. 3, 30days Kaplan-Meier survival curves showed different among SpO2 subgroups(p = 0.025) and the lowest death risk presented in the subgroup of 92 < SpO2 ≤ 96%.

**Figure 4:**
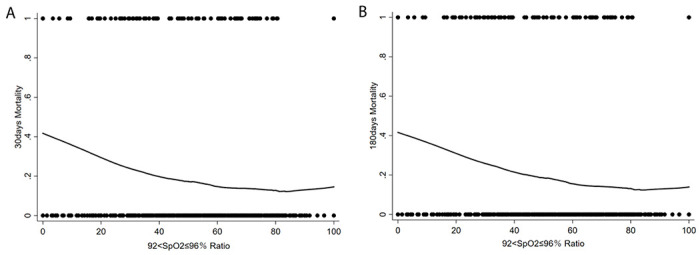
A: Association between the percentage of SpO2 counts in 92<SpO2≤96% ratio and 30days hospital mortality. Figure 4B: Association between the percentage of SpO2 counts in 92<SpO2≤96% ratio and 180days hospital mortality.

As shown in Table 3, univariate and multivariate logical analysis indicated that age(p = 0.007), BMI(p = 0.048), SOFA score(p < 0.001) and shock(p = 0.018) were predictors for 30days mortality. Table 4 described the association of the percentage of SpO2 counts in 92–96% during ICU stays and 30days/180days mortality. After adjusting for independent risk factors exhibited in Table 3, including age, BMI, SOFA, and shock, individually or together(Model1 = No adjust; Model2 = Adjust for Age and BMI; Model3 = Model2 + SOFA; Model4 = Model3 + Shock), the ratio of SpO2 in 92–96% was negatively correlated with 30days and 180days mortality(30days: Model1 OR 0.982, 95%CI 0.972–0.993; Model2 OR 0.985, 95%CI 0.974–0.995; Model3 OR 0.988, 95%CI 0.977–0.999; Model4 OR 0.988, 95%CI 0.977–0.999; 180days: Model1 OR 0.982, 95%CI 0.972–0.992; Model2 OR 0.984, 95%CI 0.974–0.994; Model3 OR 0.987, 95%CI 0.976–0.997; Model4 OR 0.987, 95%CI 0.976–0.997 ). Figure 4(A and B) further confirmed above points by LOWESS curves.

## Discussion

Our results revealed that among critically ill AECOPD patients, there was a U-shape association between SpO2 and 30days mortality. The flattest part of this U-shape line with the lowest 30days mortality was SpO2 92 to 96%. In this range, 30days and 180days mortality were lower despite higher SOFA scores and higher invasive ventilator ratio. Meanwhile, the higher rate of SpO2 falling in above range during their ICU stay, the lower 30days and 180days mortality.

Up to now, the most precise way of measuring blood oxygenation is PaO2 and SaO2, which can only be obtained by blood-gas. The invasive, expensive, and time-consuming make this approach difficult to be an appropriate way to assess blood oxygenation globally, especially in some developing country. SpO2, obtained by an instrument called a pulse oximeter, does not have any above disadvantages. Simplicity, speed, non-invasion and repeatability make it more medically practical than the former two methods. An authoritative study has proved that the measurement results of an oximeter can accurately reflect the blood oxygenation once SaO2 is greater than 75%.^3^ Meanwhile, it is hard to set a suitable level of therapeutic FiO2 without PaCO2 increase for even low concentrations (FiO2 24–28%) could increase PaCO2.^23–25^ In turn, this implies that titration of oxygen therapy to improve hypercapnia, while avoiding unnecessarily high blood oxygenation, might be preferred to the routine of fixed low FiO2.^16^ All the above reasons drive our research to focus on SpO2.

Combined with previous research, SpO2 does not conform to a normal distribution, so it was reasonable to select the median of Spo2 as a summary statistic to reflect the level of Spo2 during ICU stays.^8,26^ In addition, to reduce the error caused by reflecting SpO2 level during ICU stays only by the median, we further analyzed the relationship between the proportion of measurements within the optimal SpO2 range during ICU stays and 30days/180days mortality.^8^

It is well known that the supply of oxygen is important to human bodies and inadequate oxygen supplies can hit critically ill patients hard and lead to poor outcomes.^27^ The harmful effects of a reduction of oxygen are well known and alerting, however, the negative effects of an excess of oxygen are rarely considered.^28^ Hyperoxemia may aggravate oxidative stress and inflammatory response, thereby worsening organ function.^29^ Especially for AECOPD patients, excess oxygen supplementation often leads to recurrence or exacerbation of hypercapnia.^7,11,12,16^ However, numerous studies and questionnaires have demonstrated that patients, even with hypercapnic respiratory failure, were intended to be given high concentrations of oxygen by the majority of healthcare professionals. An entrenched culture of administering more oxygen to breathless patients may have been responsible for the ongoing practice of routine delivery of high flow oxygen.^11,12,30–32^

Several studies showed, compared with liberal oxygen therapy, the strategy of titrating oxygen into a suitable range appeared to be feasible and safe and was associated with a decrease in pulmonary atelectasis, reduced ventilator durations and lower mortality.^33–35^ It is recommended in GOLD to control oxygen therapy titrated to achieve oxygen saturation of 88–92% substantially for AECOPD patients.^5^ However, this range was traced back to a study that included only emergency transport patients, and the setting of the range had not been systematically selected, all above made scholars believe that there may be a more appropriate oxygen saturation range for AECOPD patients, especially those with critical illness and need ICU admission.^12,16^ For this issue, Ringbaek and colleagues reported for the majority of AECOPD patients received inappropriate oxygen therapy(oxygen saturation ≥ 92%) in the ambulance, the ratio of ventilatory support, the length of hospital stay, and mortality were low.^12^ A high quality retrospective study using large ICU data demonstrated that the lowest mortality at SpO2 within 94–98%, with oxygen saturation below 94 associated with a higher mortality rate, consistent with BTS.^8,13^ Although this study ruled out patients with hypercapnic respiratory failure, it suggested that higher oxygen supply may be significant for critically ill patients. A clinical trial with a large sample size conducted by handling oxygenation targets in ICU demonstrated that a lower oxygen target did not lead to lower mortality for patients in ICU.^14^ Douin et al. found that multiple non-cardiopulmonary comorbidities was a protective factor for mortality in severe trauma patients receiving high-concentration oxygen therapy.^36^ Of a similar view, evidence from several clinical studies existed that in vasodilatory and hemorrhagic shock, higher oxygen supplements may be beneficial due to the effect of hyperoxia-induced vasoconstriction.^37–39^ In addition, for patients with sepsis, a higher oxygen supplement was advocated by some authors for its potential effects of anti-hypotension, improving peripheral microvascular flow, attenuating tissue apoptosis and enhancing organ function.^28,40,41^ All above indicated that for critically ill AECOPD patients, the most appropriate oxygen saturation range may be higher than we have been thinking.

Our study provides that, once ventilated, a target range of 92–96% may be more appropriate for patients with AECOPD with hypercapnic respiratory failure than the range of 88–92% and above 96%. The range of 92–96%, where patients with older age, higher SOFA scores, higher shock rate, meaning worse general conditions, had a lower short-term and long-term mortality than the range of 88–92%. However, a higher intubation rate was found in this range. Consistent with our results, some studies have described that the risk of tracheal intubation is higher when oxygen saturation was maintained over SpO2 92%.^42,43^ But there was no difference in the duration of invasive ventilator among three SpO2 subgroups, as well as in the ratio and duration of a ventilator. The length of ICU durations in the subgroup of 92–96% is longer than 88–92%, which may be caused by unequal short-term mortality, After adjusting for death outcomes, there were no statistically significant differences in ICU durations among the three subgroups. Our study found that a higher ratio of acidosis existed in non-survival group. A higher likelihood of acidosis was found in patients with hypercapnic respiratory failure treated with higher oxygen therapy by previous observations,^16,43^ while in our research, the subgroup of 92–96%, with a higher oxygen saturation level than 88–92%, did not present a higher risk of acidosis. Our study also showed that the higher the percent of SpO2 in 92–96% during ICU stay, the lower 30days/180days mortality rate. This suggested that the SpO2 within 92 to 96% has a better clinical benefit than 88–92% and > 96%.

There are some points about our study that need further clarifications. Some studies have demonstrated that hemodynamic instability will bring weakened SpO2 monitoring signal, further leading to inaccurate measurement data.^44^ However, it is difficult to identify hemodynamic instability in MIMIC IV Database. Therefore, the use of vasoactive drugs and diagnosis of shock were incorporated into our analysis model as a criterion of hemodynamic instability. And in order to rule out factors that contribute to mortality, SOFA scores and age were also included as variables. In addition, for critically ill patients, measured SpO2 values would be left biased compared with normal states. Therefore, although the oxygen saturation range obtained by our study was suitable for critically ill AECOPD patients, it may not be appropriate for all AECOPD patients, and more systematic clinical studies are needed to further verify this SpO2 range. As mentioned above, for blood oxygen levels, FiO2 is not as intuitive as SpO2. Therefore, the data missing FiO2 was not be discarded, instead filled with logical regression method.

Although the sample size is large, allowing for more sensitivity analyses to make the results credible, limitations still existed. First of all, our subjects were taken from the same database, which was not strictly multicenter and bound to bring inaccurate results. Secondly, the oxygen saturation range of some patients may not be titrated, but difficult to increase. Albeit we adjusted the severity of patients through SOFA scores, this deviation was hard to erase. Thirdly, the data of lung function were missing because the subjects were in critical condition when they were admitted to hospital, and it was difficult to cooperate with lung function. Finally, because the results of PaCO2 can only be measured by blood gas, and most patients in the database did not get regular blood gas measurements, clinical outcomes related to PaCO2 were not included in the analysis after weighing, which will make our story incomplete.

## Conclusion

For critically ill patients with AECOPD and hypercapnic respiratory failure, a target SpO2 range of 92–96% may be more appropriate than 88–92% and > 96%. To further explore this SpO2 range, a prospective study with more oxygen target subgroups are essential.

## Figures and Tables

**Table 1:** Comparisons of demographics between survivors and non-survivors

	Total(n = 533)	Survivors(n = 445)	Non-Survivors(n = 88)	P Value
Age(years,±SD)	71.9 ± 10.4	71.4 ± 10.3	75.2 ± 10.3	0.002
Male(n%)	262(49.2)	219(48.1)	43(57.9)	0.952
BMI(kg/m2,±SD)	29.1 ± 8.5	29.4 ± 8.6	27.5 ± 8.0	0.053
Ethnicity(n%)				0.255
white	348(65.3)	297(65.3)	51(57.9)	
black	33(6.2)	31(6.8)	2(2.3)	
asian	13(2.4)	11(2.4)	2(2.3)	
others	139(26.1)	106(23.3)	33(37.5)	
ICU duration(days, IQR)	3.6(1.9–6.9)	3.5(1.8–6.5)	4.0(2.0–8.3)	0.147
ICU/Hospital duration Ratio(%,IQR)	46.9(22.7–73.8)	42.8(21.5–63.3)	91.9(51.6–100)	<0.001
SOFA(IQR)	6(4–9)	6(4–8)	8(5–11)	<0.001
Non-invasive ventilator use (n%)	227 (42.6)	196(44.0)	31(35.2)	0.126
Non-invasive ventilator duration(hours, IQR)	60(29–127)	57(27–122)	70(36–156)	0.081
Invasive ventilator use (n%)	295(55.3)	240(52.7)	55(62.5)	0.140
Invasive ventilator duration(hours, IQR)	49(24–118)	46(21–107)	85(41–170)	0.001
At ICU admission				
PaO2(mmHg, IQR)	50(37–71)	50(37–71)	46(34–73)	0.422
PaCO2(mmHg, IQR)	63(52–80)	62(52–79)	66(56–83)	0.092
FiO2(%,IQR)	50(40–100)	50(40–80)	55(40–100)	0.095
SpO2(%,IQR)	88(68–95)	88(70–95)	88(62–95)	0.633
PaO2/FiO2(mmHg, IQR)	105(71–160)	109(72–162)	94(59–151)	0.097
During ICU stay				
PaO2(mmHg, IQR)	73(54–91)	73(53–90)	75(54–92)	0.907
PaCO2(mmHg, IQR)	53(47–64)	53(47–64)	54(45–65)	0.999
FiO2(%,IQR)	40(35–50)	40(35–50)	45(40–60)	<0.001
SpO2(%,IQR)	95(94–97)	95(94–97)	95(94–97)	0.441
PaO2/FiO2(mmHg, IQR)	168(121–228)	172(124–229)	148(105–193)	0.005
Comorbidities(n%)				
Acidosis	141(26.5)	107(23.5)	34(38.6)	0.005
Congestive heart failure	257(48.2)	213(46.8)	44(50.0)	0.714
Myocardial infarction	120(22.5)	101(22.2)	19(21.6)	0.820
Shock	123(23.1)	87(19.1)	36(40.9)	<0.001
Hypertension	105(19.7)	86(18.9)	19(21.6)	0.625
Cerebrovascular disease	44(8.3)	37(8.1)	7(7.9)	0.911
Diabetes	156(29.3)	134(29.5)	22(25.0)	0.229
Renal disease	113(21.2)	97(21.3)	16(18.2)	0.448

Continuous data are presented as mean ± standard deviation (SD) or median (interquartile ranges), and categorical data are presented as frequency (percentage).

Abbreviations: SOFA, Sequential organ failure assessment; BMI, body mass index; PaO2, partial pressure of oxygen; SaO2, arterial oxygen saturation; FiO2, fraction of inspiration oxygen; SpO2, peripheral capillary oxygen saturation.

**Table 2:** Comparisons of clinical outcomes among different median ICU SpO2 subgroups

	88 ≤ SpO2 ≤ 92%(n = 59)	92 < SpO2 ≤ 96%(n = 309)	SpO2 > 96%(n = 165)	P value
Age(years, ±SD)	69.6 ± 9.1	71.3 ± 10.5	74.1 ± 10.2	0.004
Male(n%)	31(52.5)	143(46.2)	88(53.3)	0.294
BMI(kg/m2, ±SD)	29.1 ± 13.7	29.8 ± 8.7	27.6 ± 8.3	0.029
Ethnicity(n%)				0.092
white	41(69.5)	206(66.7)	101(61.2)	
black	4(6.7)	18(5.8)	11(6.7)	
asian	1 (1.7)	5(1.6)	7(4.2)	
others	13(22.0)	80(25.9)	46(27.9)	
SOFA(IQR)	5(3–7)	6(4–9)	6(4–9)	0.016
30days Mortality(n%)	14(23.7)	40(12.9)	34(20.6)	0.029
180days Mortality(n%)	15(25.4)	42(13.6)	37(22.4)	0.014
ICU duration(days, IQR)	2.9(1.6–5.3)	3.5(1.8–7.1)	37(2.1–7.6)	0.043
ICU duration(days, IQR, adjust for mortality)	27(1.7–5.3)	3.6(1.8–67)	3.6(2.0–7.6)	0.109
ICU/Hospital duration Ratio(%,IQR)	48.8(25.3–78.4)	45.8(22.9–73.1)	51.3(21.2–76.5)	0.733
Non-invasive ventilator use (n%)	38(64.4)	135(43.7)	54(32.7)	<0.001
Non-invasive ventilator duration(hours, IQR)	60(27–112)	59(29–129)	60(30–132)	0.420
Invasive ventilator use (n%)	18(30.5)	169(54.7)	108(65.5)	<0.001
Invasive ventilator duration(hours, IQR)	41(26–82)	50(23–107)	59(24–143)	0.410
Acidosis(n%)	16(27.1)	80(25.9)	45(27.3)	0.941
Shock(n%)	9(15.3)	61(19.7)	53(32.1)	0.003

Continuous data are presented as mean ± standard deviation (SD) or median (interquartile ranges), and categorical data are presented as frequency (percentage).

Abbreviations: BMI, body mass index; SOFA, Sequential organ failure assessment.

**Table 3:** Univariate and multivariate logistic analysis of risk factors and 30days mortality

	Univariate logistic analysis		Multivariate logistic analysis	
Variable	OR(95%CI)	P Value	OR(95%CI)	P Value
Age	1.04(1.01–1.06)	0.004	1.03(1.00–1.06)	0.007
Male	0.95(0.58–1.57)	0.841		
BMI	0.97(0.94–1.01)	0.122	0.97(0.94–0.99)	0.048
Ethnicity	0.95(0.67–1.33)	0.748		
SOFA	1.12(1.05–1.21)	0.010	1.13(1.06–1.21)	0.001
Acidosis	1.59(0.94–2.68)	0.083		
Congestive heart failure	1.23(0.73–2.07)	0.435		
Myocardial infarction	0.83(0.45–1.52)	0.550		
Shock	1.88(1.06–3.34)	0.031	1.96(1.12–3.43)	0.018
Hypertension	1.06(0.58–1.96)	0.847		
Cerebrovascular disease	0.85(0.35–2.06)	0.718		
Diabetes	0.97(0.54–1.75)	0.914		
Renal disease	0.66(0.33–1.30)	0.229		
Non-invasive ventilator use	0.71(0.13–4.11)	0.712		
Invasive ventilator use	0.59(0.11–3.39)	0.561		

Abbreviations: BMI, body mass index; SOFA, Sequential organ failure assessment.

**Table 4:** Logistic analysis of 92 < SpO2 ≤ 96% ratio and 30days or 180days mortality

92 < SpO2 ≤ 96% ratio and 30days mortality			
	OR	95%CI	P value
Model1	0.982	0.972–0.993	0.001
Model2	0.985	0.974–0.995	0.005
Model3	0.988	0.977–0.999	0.026
Model4	0.988	0.977–0.999	0.028
92 < SpO2 ≤ 96% ratio and 180days mortality			
	OR	95%CI	P value
Model1	0.982	0.972–0.992	< 0.001
Model2	0.984	0.974–0.994	0.003
Model3	0.987	0.976–0.997	0.014
Model4	0.987	0.976–0.997	0.015

Adjusted covariates: Model1 = No adjust; Model2 = Adjust for Age and BMI; Model3 = Model2 + SOFA; Model4 = Model3 + Shock.

Abbreviations: SOFA, Sequential organ failure assessment; OR, odd ratio; CI, confidence interval.

## Data Availability

The datasets used and analyzed during the current study are available from the corresponding author on reasonable request.
